# No Resistance Plasmid in *Yersinia pestis,* North America

**DOI:** 10.3201/eid1605.090892

**Published:** 2010-05

**Authors:** David M. Wagner, Janelle Runberg, Amy J. Vogler, Judy Lee, Elizabeth Driebe, Lance B. Price, David M. Engelthaler, W. Florian Fricke, Jacques Ravel, Paul Keim

**Affiliations:** Northern Arizona University, Flagstaff, Arizona, USA (D.M. Wagner, J. Runberg, A.J. Vogler, J. Lee, P. Keim); Translational Genomics Research Institute, Flagstaff (E. Driebe, L.B. Price, D.M. Engelthaler, P. Keim); University of Maryland School of Medicine, Baltimore, Maryland, USA (W.F. Fricke, J. Ravel)

**Keywords:** Multidrug resistance, plague, North America, Salmonella, Yersinia pestis, bacteria, plasmid, letter

**To the Editor:** Plague, caused by *Yersinia pestis*, is now largely controlled by improved sanitation and the use of antimicrobial drugs. However, before the widespread availability of antimicrobial drugs, an estimated >200 million persons died during pandemics (*1*). Today, if *Y. pestis* were to acquire antimicrobial drug resistance determinants, plague could again be a deadly disease.

Antimicrobial drug resistance in *Y. pestis* has been documented for only a few strains. The best available information is for 2 strains isolated in Madagascar in 1995 (*2*), in which resistance was conferred by plasmids not typically found in *Y. pestis*. Strain 16/95 was resistant to streptomycin only; this resistance was mediated by plasmid pIP1203 (*3*). Strain 17/95 was resistant to 8 antimicrobial drugs, including some commonly used to treat plague, such as streptomycin, tetracyclines, and sulfonamides (*2*). Multidrug resistance in 17/95 was mediated by plasmid pIP1202 (*4*). Both plasmids could be transferred by conjugation from the source *Y. pestis* strains to other *Y. pestis* strains and *Escherichia coli* (*3*,*4*). pIP1203 could be transferred from *E. coli* to *Y. pestis* in the midgut of co-infected rat fleas (*Xenopsylla cheopis*), common vectors of plague (*5*).

Comparative sequence analysis has indicated that pIP1202 shares an almost identical IncA/C backbone with multidrug-resistant (MDR) plasmids from *Salmonella enterica* serotype Newport SL254 and *Yersinia ruckeri* YR71, suggesting recent acquisition from a common ancestor (*6*). In this study, this backbone was detected in numerous MDR enterobacterial pathogens (e.g., *E. coli*, *Klebsiella* spp., and multiple *Salmonella* serotypes) isolated from retail meat products. Many of these plasmids transferred at high rates to a plasmid-free *Y. ruckeri* strain, indicating the ability to efficiently transfer among species. Meat products examined in that study originated from 9 US states, including western plague-endemic states such as California, Colorado, New Mexico, and Oregon.

To determine whether the IncA/C plasmid backbone previously found in MDR *Y. pestis* and other species exists in *Y. pestis* isolates from western U.S. states, we screened *Y. pestis* DNA. The 713 isolates were collected from from humans, small mammals, and fleas in 14 of the 17 western plague-endemic states (Table), including all states that reported human cases during 1970–2002 (*7*). We used Primer Express software (Applied Biosystems, Foster City, CA, USA) to design a TaqMan-MGB single-probe assay to detect *repA*, a plasmid replication gene present in the IncA/C plasmid backbone. We based this assay on the *repA* assay described by Carrattoli et al. (*8*) and used the same forward primer but a different reverse primer and an additional probe to facilitate screening on a real-time PCR platform.

**Table:** *Yersinia pestis* isolates collected from humans, small mammals, and fleas, United States

State	No. isolates	Years collected
Arizona	151	1975, 1977–1984, 1986–1989, 1992–1996, 1998, 2000–2002
California	129	1943, 1962, 1970, 1977, 1979–1980, 1983–1999
Colorado	97	1963, 1968, 1989, 1992, 1995–1997, 1999–2002
Idaho	2	1987, 1997
Kansas	17	1997, 1999
Montana	11	1987, 1992–1993
North Dakota	2	1986, 1993
New Mexico	124	1950, 1976–1977, 1979–1988, 1991–1992, 1994–1995, 1997–2002
Nevada	36	1980–1985, 1987, 1992–1995
Oregon	18	1959, 1970–1971, 1977, 1979, 1981–1984, 1987
Texas	5	Unknown
Utah	55	1965, 1981–1984, 1991–1995, 1999–2001
Washington	2	1984
Wyoming	64	1978, 1980, 1982–1983, 1985–1987, 1989–1990, 1997, 2000–2001

Real-time PCRs were conducted in 10-μL reaction mixtures that contained 900 nmol/L of forward (5′-GAGAACCAAAGACAAAGACCTGGA-3′) and reverse (5′-TGGCCGGAGATTCAATGATC-3′) primers, 200 nmol/L of the *repA*-specific probe (5′-6FAM-AGACTCACCGCAAATG-3′), 1× AB TaqMan Universal PCR Master Mix with AmpErase UNG (uracil N-glycosylase) (Applied Biosytems), and 1 μL of template. Thermal cycling was performed on an Applied Biosystems 7900 HT sequence detection system under the following conditions: 50°C for 2 min, 95°C for 10 min, and 40 cycles of 95°C for 15 s and 60°C for 1 min. DNA extracts from *Y. pestis* strain 17/95 (*4*) and *Salmonella enterica* serotype Newport strain SL254 (*6*) were used as positive controls.

Of the 713 *Y. pestis* isolates screened, none was positive for the IncA/C plasmid backbone, indicating that MDR as mediated by pIP1202-like MDR plasmids described by Welch et al. (*6*) was not in these samples. This finding is encouraging with regard to public health. However, we screened only for the plasmid backbone; MDR genes may have been in some of these samples but not carried by pIP1202-like MDR plasmids, especially considering that plasmids can be readily integrated into the *Y. pestis* chromosome (*1*).

Could MDR *Y. pestis* arise in North America by acquisition of an MDR plasmid, such as pIP1202, from food-animal production activities in plague-endemic regions? If so, *Salmonella* spp. would be a likely MDR plasmid donor for several reasons. First, *Y. pestis* has several plasmids that are highly similar to those in *Salmonella* spp., indicating active transfer of plasmids between these 2 bacterial groups (*6*). Second, fleas that are common vectors of plague have been shown to be naturally co-infected with *Salmonella* spp. and *Y. pestis* and capable of transmitting both organisms to rodent hosts (*9*). Third, MDR plasmids are readily transferred to *Y. pestis* in the flea gut (*5*). Fourth, transferable MDR plasmids are common among *Salmonella* spp. isolates in US food animals (*10*). Given these linkages, the transfer of an MDR plasmid from *Salmonella* spp. to *Y. pestis* seems possible. However, we emphasize that to date no evidence supports this type of event.
